# Supplementary material for: An Examination of Serum Acylcarnitine and Amino Acid Profiles at Different Time Point of Ketogenic Diet Therapy and Their Association of Ketogenic Diet Effectiveness
Source: Nutrients. 2020 Dec 23;13(1):21. doi: 10.3390/nu13010021 (PMC7822492; doi:10.3390/nu13010021)
Supplement: Supplementary file 1 [file nutrients-13-00021-s001.pdf]

Supplementary Materials

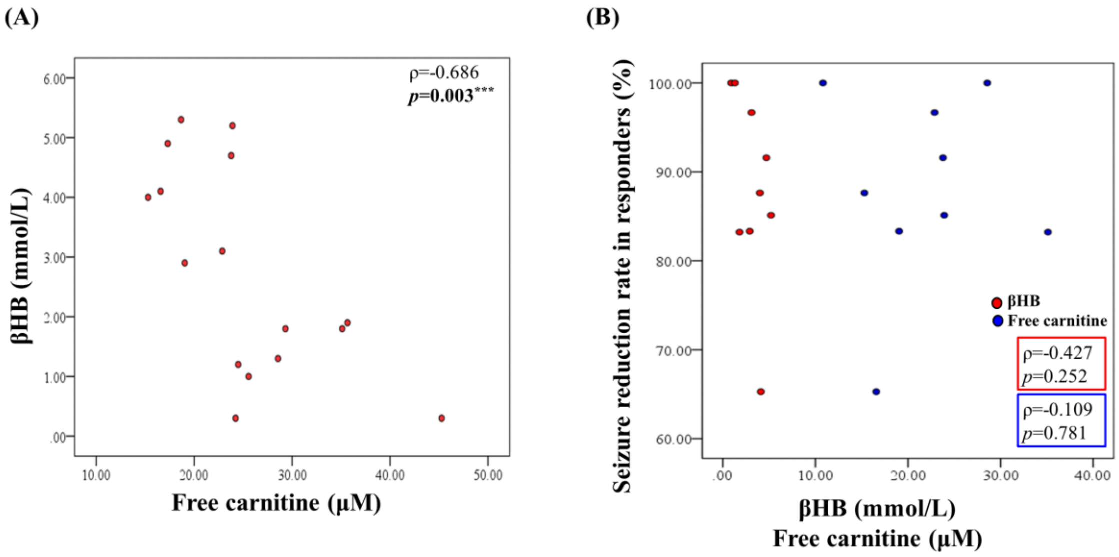

Figure S1. The correlation of free carnitine betweenβHB, and seizure reduction rate .(A) The plasma free carnitine was negatively correlated with βHB level (ρ=0.478, *P*=0.028). (B) Neither βHB nor free carnitine level was correlated with seizure reduction rate (βHB v.s. seizure reduction rate , ρ=-0.427, *P*=0.252; free carnitine v.s. seizure reduction rate, ρ=-0.109, *P*=0.781).

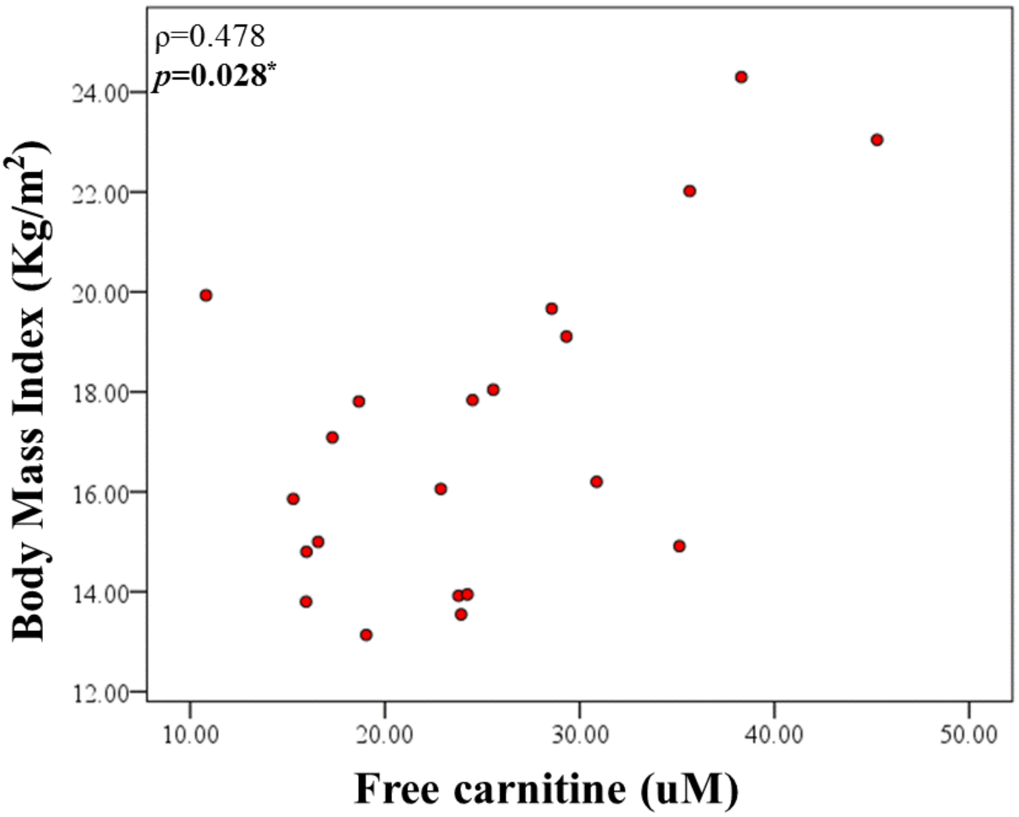

Figure 2. The plasma free carnitine was positively correlated with body mass index by Spearman zero order correlation (ϱ=0.478, *P*<0.05).

Table 1. Demograohic data of 22 study subjects.

Case	Gender	Initial age of KD (years; months)	No of AEDs	Main Seizure Type	Etiology	Baseline Seizure Frequency (per month)	12 month-Seizure Frequency (per month)	Seizure Reduction Rate
1	M	0; 8	2	Infantile spasm	HIE	5	0	≥90%
2	M	2; 4	4	Infantile spasm	HIE	476	145	50%~90%
3	M	19; 1	2	Generalized tonic-clonic	HIE	1	2	≦25%
4	M	14; 5	3	Focal motor	Immune	33	19	25%~50%
5	M	10; 10	2	Generalized tonic-clonic	Unknown	1	1	≦25%
6	F	15; 5	4	Generalized tonic-clonic	Unknown	1	0	≥90%
7	M	2; 7	3	Infantile spasm	HIE	252	88	50%~90%
8	M	10; 3	3	Focal motor	Unknown	224	33	50%~90%
9	F	13; 2	2	Infantile spasm	Unknown	280	222	≦25%
10	F	15; 11	2	Generalized tonic-clonic	Genetic	5	1	50%~90%
11	M	3; 1	3	Generalized tonic-clonic	Genetic	224	19	≥90%
12	M	11; 4	2	Focal motor	HIE	3	1	50%~90%
13	M	9; 11	2	Generalized tonic-clonic	Genetic	32	18	25%~50%
14	M	4; 1	4	Focal motor	Immune	36	13	50%~90%
15	M	1; 3	2	Infantile spasm	Genetic	70	37	25%~50%
16	M	17; 2	1	Focal motor	Genetic	1	0	≥90%
17	M	1; 2	3	Generalized tonic-clonic	Structural	168	28	50%~90%
18	F	0; 10	4	Focal motor	Structural	30	80	≦25%
19	F	2; 4	2	Focal motor	Genetic	8	4	50%~90%
20	F	9; 7	3	Focal motor	Infectious	1	2	≦25%
21	F	10; 1	3	Focal motor	Genetic	55	24	50%~90%
22	F	0; 3	4	Generalized tonic-clonic	Structural	51	33	25%~50%

HIE: Hypoxic-ischemic encephalopathy.

Table 2. The clinical characteristics of responders and non-responders. Seizure types and etiologies were presented as the number (percentage). Patient profiles, including age, body weight (BW), and body mass index (BMI) are presented as the mean ± standard deviation (SD). The number of AEDs used is presented as the median ± SD. Groups differences were analyzed by the Fisher's exact test or repeated measures one-way analysis of variance with the Tukey method for post hoc comparisons**.** *R*: responders; *NR*: non-responders; **P*<0.05. All statistically significant values are bolded. **P*<0.05.

	**Visit 1 Initial**			**Visit 2 3 month**			**Visit 3 6 month**			**Visit 4 9 month**			**Visit 5 12 month**		
	**R (N=13)**	**NR (N=9)**	***p***	**R (N=13)**	**NR (N=9)**	***p***	**R (N=13)**	**NR (N=9)**	***p***	**R (N=13)**	**NR (N=9)**	***p***	**R (N=13)**	**NR (N=9)**	***p***
**Seizure types,no (%)**															
Focal motor	5 (42.7)	4 (40.0)	0.841												
GTC	4 (33.3)	4 (40.0)	0.761												
Infantile spasm	3 (25.0)	2 (20.0)	0.792												
**Etiology,no (%)**															
Structural	1 (8.3)	2 (20.0)	0.469												
Genetic	4 (33.3)	3 (30.0)	0.875												
Infectious	0 (0.0)	1 (10.0)	0.284												
Metabolic	0 (0.0)	0 (0.0)	1												
Immune	1 (8.3)	1 (10.0)	0.899												
HIE	4 (33.3)	1 (10.0)	0.212												
Unknown	2 (16.7)	2 (20.0)	0.85												
**Age** Mean (SD)	7.84 (5.78)	11.00(5.21)	0.207												
**BW (Kgs)** Mean±SD	22.18 ±22.62	29.70±18.04	0.406	24.56±19.95	29.87 ±17.24	0.516	25.03±21.04	28.94±16.13	0.641	19.36±11.24	28.84±15.56	0.08	21.16±11.76	32.13±14.54	0.053
**BMI (Kg/m^2)** Mean±SD	16.26±4.14)	19.14±3.31	0.057	16.62±3.86	18.73±3.00	0.129	16.36±3.33)	17.89±2.94)	0.231	15.16±2.00)	17.40±3.08	**0.042***	15.48±2.16	18.23±3.25	**0.025***
**No. of AEDs** Median	3	2	0.366	3	2	0.212	3	2	0.147	3	2	0.556	3	2	0.326
**AEDs (mono or add-on), no (%)**															
Valproate	2 (9.09)	6 (27.27)	**0.029***	2 (9.09)	6 (27.27)	**0.029***	2 (9.09)	6 (27.27)	**0.029***	2 (9.09)	6 (27.27)	**0.029***	2 (9.09)	6 (27.27)	**0.029***
Oxcarbazepine	4 (18.18)	2 (9.09)	0.434	4 (18.18)	2 (9.09)	0.434	4 (18.18)	2 (9.09)	0.434	4 (18.18)	2 (9.09)	0.434	4 (18.18)	2 (9.09)	0.434
Topiramate	2 (9.09)	2 (9.09)	0.901	2 (9.09)	2 (9.09)	0.901	2 (9.09)	2 (9.09)	0.901	2 (9.09)	2 (9.09)	0.901	1 (4.55)	2 (9.09)	0.485
Lamotrigine	1 (4.55)	2 (9.09)	0.485	1 (4.55)	2 (9.09)	0.485	1 (4.55)	2 (9.09)	0.485	1 (4.55)	2 (9.09)	0.485	1 (4.55)	2 (9.09)	0.485
Vigabatrin	2 (9.09)	1 (4.55)	0.626	2 (9.09)	1 (4.55)	0.626	2 (9.09)	1 (4.55)	0.626	2 (9.09)	1 (4.55)	0.626	2 (9.09)	1 (4.55)	0.626

R: Responders; NR: Non-responders;*P<0.05; **P<0.01;***P<0.005; ****P<0.001

Table S3. The raw data of metabolic parameters in responders and non-responders for serial follow-up.

	Visit 1 Initial			Visit 2 3 month			Visit 3 6 month			Visit 4 9 month			Visit 5 12 month		
	R (N=13)	NR (N=9)	*p*	R (N=13)	NR (N=9)	*p*	R (N=13)	NR (N=9)	*p*	R (N=13)	NR (N=9)	*p*	R (N=13)	NR (N=9)	*p*
**Average BHB (mmol/L)**	3.24±1.00	2.33±1.91	0.302	3.18±1.82	2.06±1.04	0.098	3.45±1.62	1.69±1.72	**0.020***	2.63±1.70	1.93±1.36	0.306	2.47±1.67	2.21±2.10	0.788
**Average free carnitine (μM)**	25.12±7.28	26.20±10.69	0.802	22.93±4.65	25.10±10.24	0.571	20.84±6.89	29.03±8.86	**0.026***	20.85±4.72	24.84±8.57	0.225	24.16±5.79	23.66±10.07	0.898
**C2**	22.23±9.87	16.91±4.99	0.11	28.42±7.74	27.85±8.13	0.875	29.49±11.85	29.94±5.14	0.908	26.17±9.66	25.35±9.64	0.85	31.48±14.92	23.00±6.01	0.134
**C3**	1.32±0.43	2.36±1.27	**0.024***	0.80±0.68	1.79±1.49	0.074	0.72±0.48	1.73±1.08	**0.012***	0.64±0.32	1.47±1.03	**0.026***	0.95±0.54	1.41±1.041	0.243
**C4OH**	0.15±0.12	0.07±0.03	**0.033***	0.39±0.14	0.33±0.13	0.332	0.41±0.20	0.36±0.13	0.521	0.35±0.19	0.29±0.05	0.326	0.38±0.25	0.28±0.14	0.304
**C4-DC**	0.41±0.17	0.56±0.27	0.154	0.14±0.08	0.30±0.28	0.113	0.14±0.12	0.25±0.24	0.227	0.14±0.11	0.26±0.25	0.194	0.15±0.16	0.22±0.19	0.411
**C5**	0.11±0.04	0.13±0.08	0.582	0.08±0.03	0.16±0.08	**0.019***	0.07±0.03	0.17±0.05	**0.000******	0.07±0.03	0.17±0.06	**0.001*****	0.08±0.03	0.12±0.06	0.051
**C5:1**	0.41±0.17	0.02±0.01	**0.007*****	0.01±0.01	0.02±0.01	0.197	0.02±0.01	0.02±0.01	0.356	0.01±0.00	0.02±0.01	0.195	0.01±0.01	0.02±0.01	0.206
**C6**	0.08±0.03	0.10±0.12	0.607	0.06±0.02	0.12±0.13	0.139	0.06±0.02	0.06±0.02	0.708	0.05±0.01	0.25±0.13	0.229	0.07±0.03	0.05±0.02	0.176
**C8**	0.09±0.04	0.12±0.13	0.517	0.07±0.04	0.16±0.13	0.08	0.07±0.03	0.09±0.03	0.349	0.06±0.02	0.11±0.04	**0.001*****	0.09±0.04	0.09±0.03	0.97
**C10**	0.10±0.06	0.14±0.14	0.527	0.09±0.05	0.19±0.10	**0.006****	0.10±0.05	0.13±0.05	0.132	0.07±0.02	0.12±0.05	**0.005****	0.12±0.06	0.12±0.05	0.874
**C10:1**	0.07±0.03	0.08±0.03	0.77	0.08±0.04	0.10±0.04	0.13	0.09±0.06	0.09±0.04	0.9	0.05±0.02	0.10±0.04	**0.000******	0.10±0.06	0.10±0.03	0.957
**C12**	0.06±0.03	0.12±0.13	0.157	0.05±0.01	0.11±0.04	**0.000******	0.05±0.02	0.07±0.02	**0.047***	0.05±0.02	0.08±0.03	**0.019***	0.08±0.02	0.07±0.03	0.807
**C14**	0.08±0.04	0.12±0.06	0.171	0.07±0.02	0.11±0.06	0.066	0.06±0.03	0.08±0.02	**0.033***	0.05±0.02	0.09±0.04	**0.016***	0.08±0.03	0.06±0.02	0.116
**C14:1**	0.07±0.02	0.08±0.04	0.399	0.08±0.04	0.14±0.06	**0.013***	0.09±0.04	0.09±0.02	0.97	0.08±0.03	0.11±0.05	0.262	0.13±0.09	0.09±0.04	0.261
**C14:2**	0.03±0.02	0.04±0.01	0.776	0.04±0.01	0.06±0.02	**0.000******	0.04±0.03	0.04±0.01	0.569	0.03±0.02	0.05±0.02	0.142	0.06±0.03	0.05±0.02	0.459
**C16**	0.73±0.30	1.19±0.38	**0.003*****	0.56±0.20	1.36±0.84	**0.008****	0.51±0.18	1.23±0.64	**0.002*****	0.47±0.24	1.16±0.58	**0.001*****	0.61±0.21	0.83±0.33	0.103
**C16:1**	0.07±0.03	0.11±0.04	**0.013***	0.07±0.01	0.11±0.06	**0.030***	0.06±0.02	0.09±0.05	0.108	0.06±0.03	0.09±0.06	0.117	0.08±0.02	0.07±0.03	0.292
**C18**	0.48±0.19	0.68±0.20	**0.033***	0.55±0.22	1.00±0.71	0.08	0.50±0.17	1.01±0.55	**0.009****	0.46±0.19	0.92±0.40	**0.002*****	0.60±0.22	0.77±0.26	0.142
**Phenylalanine**	48.54±10.52	45.50±7.99	0.436	39.08±7.44	41.30±5.79	0.457	35.51±6.08	43.81±7.73	**0.020***	35.50±6.90	36.29±4.71	0.748	39.33±6.85	40.10±5.99	0.807
**Tyrosine**	50.94±15.63	45.39±11.43	0.328	35.81±8.67	38.16±11.81	0.64	33.56±6.21	34.48±4.65	0.723	36.15±5.74	32.77±8.81	**0.021***	32.87±3.24	37.19±8.62	0.161
**Glycine**	265.93±83.46	285.17±51.86	0.494	352.94±104.61	327.91±47.87	0.461	382.13±178.43	301.78±79.04	0.182	346.06±101.54	332.68±57.38	**0.014***	303.91±79.82	345.11±88.97	0.314
**Proline**	174.99±50.78	199.6±66.47	0.361	148.04±67.00	149.97±38.21	0.934	116.74±33.31	123.94±14.07	0.517	116.34±26.14	119.48±29.42	0.54	131.64±49.06	165.02±47.75	0.156
**Leucine**	182.87±79.60	177.07±44.63	0.822	135.50±50.25	172.45±52.14	0.122	115.84±35.13	148.87±30.62	**0.037***	116.93±29.40	139.13±41.82	0.254	119.73±23.34	151.08±43.36	0.059
**Methionine**	16.73±6.36	18.34±5.78	0.537	12.76±5.15	14.96±7.49	0.482	11.77±2.79	14.62±6.96	0.327	12.37±3.02	10.22±2.83	**0.049***	11.49±3.65	11.81±3.61	0.857

R: responders; *NR*: non-responders; **P*<0.05, ***P*<0.01, ****P*<0.005, *****P*<0.001.
